# How do oilcloth sessions work? A realist evaluation approach to exploring ripple effects in an implementation strategy

**DOI:** 10.1108/JHOM-01-2023-0022

**Published:** 2024-05-27

**Authors:** Jeanette Wassar Kirk, Nina Thorny Stefansdottir, Ove Andersen, Mette Bendtz Lindstroem, Byron Powell, Per Nilsen, Tine Tjørnhøj-Thomsen, Marie Broholm-Jørgensen

**Affiliations:** Clinical Research Department, Hvidovre Hospital, Hvidovre, Denmark; Department of Health and Social Context, National Institute of Public Health, University of Southern Denmark, Copenhagen, Denmark; Emergency Department, Hvidovre Hospital, Hvidovre, Denmark; Department of Clinical Medicine, University of Copenhagen, Copenhagen, Denmark; Center for Mental Health Services Research, Brown School, Washington University in St Louis, St Louis, Missouri, USA; Center for Dissemination and Implementation, Institute for Public Health, Washington University in St Louis, St Louis, Missouri, USA; John T. Milliken Department of Medicine, Division of Infectious Diseases, School of Medicine, Washington University in St Louis, St Louis, Missouri, USA; Department of Medicine, Health and Caring Sciences, Linköping University, Linköping, Sweden

**Keywords:** Implementation strategies, Realist evaluation, Ripple effects, Qualitative research, Ethnography, Semi-structured interviews

## Abstract

**Purpose:**

To explore the mechanisms of the implementation strategy, “oilcloth sessions” and understand and explain the ripple effects of oilcloth sessions as a strategy to implement a new emergency department.

**Design/methodology/approach:**

A qualitative design was used whereby data were collected using field notes from an ethnographic study of the oilcloth sessions and follow-up semi-structured interviews with staff, managers and key employees who participated in the oilcloth sessions. The data analysis was inspired by the realist evaluation approach of generative causality proposed by Pawson and Tilley.

**Findings:**

The primary ripple effect was that the oilcloth sessions were used for different purposes than the proposed program theory, including being used as: (1) a stage, (2) a battlefield, (3) a space for imagination and (4) a strategic management tool influencing the implementation outcomes. The results bring essential knowledge that may help to explain why and how a well-defined implementation strategy has unplanned outcomes.

**Originality/value:**

Unintended outcomes of implementation strategies are an underexplored issue. This study may help implementation researchers rethink the activities required to reduce unintended negative outcomes or explore potential unplanned outcomes and, in this way, hinder or enhance outcomes, effectiveness and sustainability. Future studies within implementation research should incorporate attention to unintended outcomes to fully understand the impact of implementation strategies.

## Introduction

Supporting the integration of evidence-based practices or programs into healthcare settings requires the development of feasible and effective implementation strategies (Eccles and Mittman, 2006), defined as methods or techniques used to enhance the adoption, implementation, and sustainability of a clinical program or practice (Proctor *et al*., 2013). More than 70 discrete implementation strategies have been identified (Powell *et al*., 2012, 2015), and there is increasing evidence of their effectiveness (Grimshaw *et al*., 2012; Powell *et al*., 2019). However, poor understanding of how and why implementation strategies work or fail have led to calls for research focusing on mechanisms (Lewis *et al*., 2018, 2020; Williams, 2016), defined as the processes or events through which an implementation strategy operates to affect one or more implementation outcomes. Failure to understand the mechanisms of implementation strategies has several consequences: (1) it hinders efforts to systematically design and tailor implementation strategies, (2) it limits our ability to learn from negative studies and replicate positive findings, (3) it prevents the successful adaptation of an implementation strategy developed in one setting in another, and (4) it limits understanding of generalizability and the role of context in implementation (Geng *et al*., 2022; Lewis *et al*., 2021).

Ideally, implementation strategies are developed and selected with a specific purpose, such as to overcome an identified barrier or to achieve a specific implementation outcome (e.g. fidelity, sustainment). Within a positivistic paradigm, we might consider the pathways between implementation strategies and the intended outcomes through a causal lens, whereas in a realist paradigm, the causal associations themselves are rarely universal; they are adaptive “demi-regularities”, understood as frequently reproduced behaviors or patterns that can be seen in human activity, and therefore always strongly determined by setting and context (Dalkin *et al*., 2015). However, implementation strategies are social processes that require behavioral changes (Neal and Neal, 2019). This may lead to unplanned, non-linear, or unexpected “ripple effects” (Lipsitz, 2012; Pullmann *et al*., 2022). Ripple effects have been discussed in the literature as being positive or negative outcomes that are caused by implementation strategies and are unintentional, unplanned, unanticipated, and/or more salient to stakeholders other than implementers and researchers (Pullmann *et al*., 2022).

Ripple effects of an implementation strategy in the form of oilcloth sessions, which is a micro-simulation method, have never been explored in the scientific literature. Oilcloth sessions as a strategy are employed in several studies focusing on organizational innovations in Denmark which pertains to the reorganization of hospitals (Madsen and Meier, 2017) and physical environments (Andersen, 2016). While Oilcloth sessions are a Danish innovation, they can be linked to international tabletop models, a prevalent method in gaming and computer animation (French and Hofstadter, 1991). It’s worth noting that Oilcloth sessions involve a relatively limited use of 3D animation compared to other approaches. In a previous study, we investigated oilcloth sessions and the challenges perceived by healthcare professionals, managers, and other key employees when they planned, conducted, and evaluated oilcloth sessions intended to facilitate the implementation of a new emergency department (ED) in Denmark (Kirk *et al*., 2022). Oilcloth sessions are expected to allow key employees and managers to be involved in the implementation process by participating in the sessions and achieving insight into the physical and material context. The assumption is that the sessions increase the managers’ and key employees’ attitudes, motivation, and responsibility for implementing the new ED. The overall purpose of the oilcloth sessions was thus to increase the implementation outcomes acceptability for a new ED and fidelity in getting the ED implemented as planned (see Table 1 for the program theory). Our results demonstrate the importance of ensuring alignment between didactic elements in the oilcloth session (e.g. objectives, suitable participants, and clinical cases) if the strategy is to have a positive outcome (Kirk *et al*., 2022). Given a lack of alignment between the selected clinical cases, the invited participants and unclear objectives, which was identified as a challenge, addressing ripple effects became a priority for practice and research.

Oilcloth sessions could be an effective implementation strategy, but there is a lack of knowledge on whether and how they work. Thus, it is important to formally explore their effectiveness in relation to the program theory (i.e. proposed causal pathways) and to examine any ripple effects that emerge from their use. In this study, we aim to (1) explore the mechanisms of oil cloth sessions and (2) identify ripple effects of oilcloth sessions.

## Theoretical framework

### What works, for whom, in what respects, to what extent, in what contexts, and how?

To explore the mechanisms and ripple effects of oilcloth sessions, we are inspired by the realist evaluation approach of generative causality proposed by Pawson and Tilley (1997a, b), where the aim is to identify the underlying generative mechanisms that explain “how” outcomes were caused and the influence of context on both mechanisms and outcomes. In realist evaluation, outcomes are conceptualized as patterns of intended and unintended consequences that emerge from the activation of mechanisms in different contexts; thus, realist evaluation represents an appropriate approach for this inquiry.

Although inspired by and closely related to the critical realist principles, Pawson and Tilley refer to scientific realism as their philosophical source of inspiration for realist evaluation (Mukumbang *et al*., 2023; Pawson and Tilley, 1997a, b). With this philosophical view, programs and participants are grounded in a stratified social reality, where human action is embedded in an interplay between individuals and institutions, each with their own interest and objectives. In this line of though, causal mechanisms reside in social relations and context as much as in individuals (Marchal *et al*., 2012), because context stipulates causal mechanisms, which in turn trigger (or do not trigger) that the causal potential is realized in form of a causal outcome (Greenhalgh and Manzano, 2022; Leeuw and Astbury, 2010). Consequently, analytical attention to different dimensions of context is central for explaining the successes and failures of social programs, including implementation strategies.

The complete realist research question is: What works, for whom, in what respects, to what extent, in what contexts, and how (Gilmore *et al*., 2019). The line of reasoning is that knowledge that is derived from these questions will improve the effectiveness of a given program or strategy. Hence, a key tenet of realist research is to explore and explain what goes on in the system that connects various inputs and outputs (Dalkin *et al*., 2015). To this end, realist evaluation seeks to identify a series of hypotheses on the interrelationship between context, mechanisms and outcomes, so-called CMO configurations (Pawson and Tilley, 1997a, b). The cumulative construction of CMO configurations, or program theories, offer a level of abstraction that transcends the implementation strategies and provide knowledge about how the strategies work in specific contexts.

### Mechanisms of change

The term “mechanism” means different things depending on the field of research and the context in which it is used (Leeuw and Astbury, 2010). With inspiration from critical realism, Pawson and Tilley's definition of mechanisms ontologically pertains to the “real” domain, which means that they are usually hidden, but can be known through empirical the empirical domain (Pawson and Tilley, 1997a, b). Thus, in realist evaluation the researcher uses the information from the empirical material to explore and deduct mechanisms (Brekke *et al*., 2019). Chen and Rossi (1987) were among the first to introduce the term “mechanism” in evaluation research. In their early work, they argued, that “the theory-driven approach avoids the pitfalls of black-box evaluation and provides better understanding of the causal mechanisms underlying the relationship between treatment and effects” (Chen and Rossi, 1987, p. 102). Weiss (1997) builds on this understanding of causal mechanisms arguing that mechanisms are the response that the program activities trigger from the recipients. Of relevance for this study, the premise of mechanisms is that it is the recipients of the implementation strategy that make it work (or not) depending on how they respond to the resources it offers them (Pawson and Tilley, 1997a, b). The process of receiving, interpreting, and acting is the mechanism that generates the change(s) in, for example, behavior or attitude (Salter and Kothari, 2014).

For Pawson and Tilley, mechanisms are identified at the level of human reasoning. The reasoning of the actors in response to the resources or opportunities provided by the program or strategy is what causes the outcome (Dalkin *et al*., 2015; Pawson and Tilley, 1997a, b). To help avoid the risk of conflating the implementation strategy with the mechanism, we draw on Dalkin *et al.*’s (2015) further development of Pawson and Tilley’s definition of mechanisms, which contributes a distinction between mechanism resource and mechanism reasoning. Their understanding of mechanisms presupposes that people change behavior as a result of a resource (e.g. an implementation strategy) being made available to them (e.g. an oilcloth session) that causes them to reflect, reason, and act in certain ways. Dalkin *et al.*'s (2015) definition of mechanism emphasizes that resources are introduced into a pre-existing context, which induces an individual’s reasoning, leading to an outcome. In this line of thought, outcomes are patterns of intended and unintended consequences (i.e. ripple effects) of the resources added. Thus, the realist research approach enables us to illustrate the ripple effects of oilcloth sessions by including the interplay between contextual factors and mechanisms, such as norms, conditions, processes, and resources, that influence the impact of the oilcloth sessions on the implementation of a new ED.

## Methods

### Study design

This study is based on a qualitative design, which is particularly suitable for studies that explore deeper understandings of underlying structures, cultural attitudes, motives and mechanisms, and any relationships and discrepancies between these (Denzin and Lincoln, 2018). The data were originally obtained for an ethnographic study exploring participants’ experiences of oilcloth sessions as a strategy when implementing new EDs (Kirk *et al*., 2022). Data included field notes and follow-up semi-structured interviews with staff, managers, and key employees who participated in the oilcloth sessions in 2019 and 2020 (Kirk *et al*., 2022; Kvale and Brinkmann, 2009). For this study, we carried out a secondary analysis of the empirical material, with a focus on the identification of underlying causal mechanisms generating unplanned outcomes of oilcloth sessions (Gilmore *et al*., 2019). In this section, we present the data collection from the ethnographic study and the secondary analysis of CMO configurations.

### Political context and setting

The public healthcare system in Denmark is funded by taxpayers and provides free treatment for primary medical care, hospitals, and homecare services for all citizens. Centralization of emergency services in Denmark has been advocated politically and is expected to improve access to specialized facilities and equipment, and to reduce the risk of being admitted to a wrong “silo” of highly specialized physicians, which increases the risk of erroneous or missed diagnoses in the first hours of acute hospitalization (Sundhedsstyrelsen, 2020). In Denmark, EDs are a primary entry point for about 1 million of the 1.3 million yearly acute hospitalizations in Denmark (Statistikbanken, 2018) and thereby are a linchpin of the acute healthcare system. The new EDs are expected to provide high quality treatment and care, enhance the physical environment, improve the coordination and interdisciplinary collaboration across specialties and improve patient-experienced quality (Kirk *et al*., 2022; Sundhedsstyrelsen, 2020).

The implementation object in this study is a new ED at a university hospital in the Capital Region of Denmark. Among other things, the new ED involves physical changes, such as new buildings and changes in the organization involving a merger between two existing EDs in the hospital, the gastroenterological ED and the general ED, which currently handles all other acute admittances including medical diseases and trauma (Stefánsdóttir *et al*., 2022).

### The implementation strategy: oilcloth sessions

The Executive Board of the hospital carried out several implementation strategies to facilitate the implementation of the new ED, including oilcloth sessions (see Appendix 1). Oilcloth sessions are a micro-simulation method, combining elements of four types of implementation strategies: educational (training), restructuring (altering professional roles, physical structures, equipment, etc.) facilitation (supporting processes) and modeling and simulation (simulating change) (Powell *et al*., 2012, 2015), with the aim of training participants in new and existing patient pathways and to introduce the new physical building (Kirk *et al*., 2022). During the oilcloth sessions, participants worked together on a blueprint of the layout of the new ED combined with plastic figures, generating knowledge, workplace learning and experiences in relation to the implementation of the new ED (Kirk *et al*., 2022).

Thirteen oilcloth sessions were facilitated by two members of the Executive Board between October 2019 and November 2020. The participants and the facilitator stood around a table where the blueprint was placed. During the sessions, the researcher, members of the Executive Board, and managers from non-clinical departments sat on chairs along the walls and could also participate (Kirk *et al*., 2022). Due to the layout of the room, it was not possible for the facilitator to move around. Therefore, the facilitator was positioned on one side of the blueprint, which was placed on the table. Throughout the process of playing through pre-defined cases, the facilitator posed open-ended questions to the group of participants. Sometimes, the questions were specifically directed at management representatives if, for example, the discussion revolved around financial matters. However, all participants had the opportunity, as a basic premise, to spontaneously contribute to the questions and answers during the session.

Oilcloth sessions were held at the beginning of the pre-implementation period, phase 1 according to the Quality Implementation Framework (QIF) (Meyers *et al*., 2012).

### Participants

Members of the Executive Board, managers of clinical departments, health professionals, and key employees from one or two other specialty departments participated in the oilcloth sessions (see Table 2 for participating departments). The managers were invited to participate by the Executive Board and were responsible for inviting key employees and health professionals from their department. A key employee is an employee appointed by managers to play a central role in the implementation of a new ED in relation to their department. On average, 15–20 participants were present at each oilcloth session (Kirk *et al*., 2022). The majority of participants attended two oilcloth sessions. Members of the Executive Board, the chief physician, and the chief nursing officer from the existing ED attended all 13 oilcloth sessions.

Because all specialty departments were affected by the implementation of a new ED, we deemed it necessary to invite everyone who participated in the oilcloth sessions to an interview (*N* = 64 including two members of the Executive Board), resulting in 53 semi-structured interviews (Kirk *et al*., 2022) (see Table 3 for positions and professions).

### Data collection

#### Participant observations and field notes

Participant observations allowed us to observe all 13 oilcloth sessions and how these were held, and to explore contextual factors (Kirk and Haines, 2020). Observations also helped interpret episodes and situations that participants verbally reported and to further understanding of the participants’ experiences (Marshall and Rossman, 1989). JWK and NTS observed the sessions with the aid of an open observation matrix divided into three columns: (1) observations; (2) reflections; and (3) analytical remarks. At the end of a session, reflections, additional notes, analytical concepts, and remarks were added. Notes, reflections and central points from the fieldnotes were continuously discussed with the rest of the author group (Kirk *et al*., 2022).

#### Semi-structured interviews

Interviews with the participants were conducted between October 2019 and December 2020 by JWK and NTS (Kirk *et al*., 2022). Interviews lasted from 26 min to 1.03 h. The semi-structured interview guide covered eight themes and was developed by NTS and JWK based on knowledge from 20+ years of experience within the hospital and previous knowledge of the field obtained by observing at meetings related to the implementation of the ED. All interviews were recorded and transcribed verbatim.

### Data analysis

The study presented in this article was not initially designed as a realist evaluation. Instead, it is a result of a secondary analysis (Payne and Payne, 2004) of the empirical material from the ethnographic study exploring participants’ experiences of oilcloth sessions (Kirk *et al.*, 2022). Attention to unintended outcomes of the implementation strategy emerged during the thematic analysis of the ethnographic study and thus gave rise to the secondary analysis with a focus on ripple effects. The secondary analysis was conducted inspired by the following research question: What works, for whom, under what circumstances, and how? Here, we identified the context, mechanisms, and outcomes (CMO configurations) that could explain the ripple effects.

For the secondary analysis, JWK read and re-read the transcribed data both from the field study and the interviews to get a sense of the entire dataset. Then data were condensed in a coding scheme, divided into meaning units and then abstracted on a manifest level close to the text and then on a latent level with interpretations using different empirical and theoretical concepts. Sub-themes and themes were defined (Graneheim and Lundman, 2004) (Appendix 2). The themes constituted four ripple effects. MBJ elicited CMO-configurations by assessing the outcome patterns for each ripple effect, making the elicitation data-driven, rather than testing pre-defined CMO-configurations. Retroductive analysis enabled the identification of how the combination of specific contextual factors and mechanisms generated various intended and unintended implementation outcomes (Gilmore *et al*., 2019). The CMO-configurations were refined and discussed by MBJ and JWK until agreement was reached. To provide a more generalizable understanding of how the oilcloth sessions operated, MBJ and JWK applied the initial program theory to compare how the identified ripple effects influenced the intended implementation outcomes. The final ripple effects and CMO configurations were discussed with the entire author group, strengthening the validity of the analysis.

### Ethical considerations

According to Danish law, formal ethical approval is not mandatory for studies that do not involve biomedical issues therefore ethical approval was not required for the study. Data approval was issued by the Capital Region (I-Suite no. VD-2019–160). The project adheres to the directives of the Helsinki Declaration (Code, 1949) by informing all participants about the aim of the study and assuring that participation in the interviews was voluntary and that they and the results would be anonymized. Written informed consent was obtained from the participants before the interviews, and each participant was given the opportunity to withdraw from the interviews, though no one chose to do so. Anonymity was achieved by assigning participants a code instead of using their full names in the field notes and the interview data. The researchers maintained a confidential file of identifiers tied to participant backgrounds so that the interview data (recordings and transcripts) could be coded as a basis for in-depth analysis.

## Results

The primary ripple effect was that the oilcloth sessions were used for different purposes than the proposed program theory, including being used as: (1) a stage, (2) a battlefield, (3) a space for imagination and (4) a strategic management tool. The results are presented pertaining to these four ripple effects, the interplay between the contextual factors and the mechanisms, and how the ripple effects affected the implementation outcomes (see Table 4 for the CMO configurations identified).

### A stage

As a refinement to the initial program theory, the analysis showed that being involved also included, that managers and key employees experienced being acknowledged and listened to by the Executive Board. The analysis showed that the managers and key employees perceived the oilcloth sessions as a stage at which they could present new ideas and raise concerns. A stage is defined as a specific action that unfolds at a particular place and time (Dictionary Cambridge). The stage concept was not directly stated in the interviews, but the fieldnotes show how managers and key employees acted and used the sessions in two ways: as a mouthpiece for their specialty vis-à-vis the Executive Board and as a showroom for all participants. One important contextual factor that activated this behavior was the presence of the members of the Executive Board at all the oilcloth sessions; several managers and trainee physicians saw this as an opportunity to present new ideas about patient pathways and collaboration that the participants considered novel but did not expect the Executive Board to be aware of them. A key employee expressed:

Ideas were brought to the table, which […] were articulated to the Executive Board […]. I think there is also a responsiveness to it, when you stand in such a room [oilcloth sessions], rather than if you just tell them: “I think this is a good idea ….”. So, I guess they might be listening in a slightly different way, right? [informant 041]

Other managers saw the opportunity to express concerns related to their specialty and the design of the new building in relation to the implementation of the new ED to the Executive Board:

I think some of the things we as managers had talked about beforehand in our department became visible; for example, a lot of concerns about the distance between the patient rooms and I had the impression that now all of a sudden there was someone else who could actually see it too, the Executive Board. [informant 037]

Oilcloth sessions were used by the managers as a direct communication channel to the members of the Executive Board, with the expectation that their ideas and concerns were heard more than in everyday practice.

The oilcloth sessions also served as a stage by offering an opportunity for the participants to be seen by a larger audience, including managers from other specialty departments and the Executive Board. According to the participants, the oilcloth sessions made it possible for them to demonstrate how they cared for and treated their patients with the “right patient attitude” from the perspectives of their specialty identity. A key employee expressed:

They [the other managers and the Executive Board] have seen our will to treat the patients well with high quality, this was what we focused on …. [informant 036]

The participants experienced receiving recognition when the Executive Board loudly acknowledged and listened to the managers' announcements and ideas. A manager explained:

I think it was good for me to say that we wanted children to be treated as equally as adults in the ED. Here this was confirmed with “yes, of course, they should” … said the Executive Board. I think it was good for me to say it directly to the Executive Board so they could hear what our priorities were. [informant 039]

Some participants’ experience of being heard and listened to, and involvement by the Executive Board positively influenced their views of both the Executive Board’s participation and their overall acceptance of the development of a new ED and the implementation process. Being acknowledged by the Executive Board also affected the internal self-image of the participating managers; they perceived themselves as well considered and with a strengthened specialty identity vis-à-vis the other managers. A manager pronounced:

I assume that the managers of the new ED can see that our ideas are useful. They are supported by the Executive Board. It must make an impression and give our specialty some respect. [fieldnotes no. 2]

Due to the Executive Board’s position as top managers and the structural power of their position, the oilcloth sessions became a stage where the participants experienced being able to voice their ideas and concerns in the presence of the members of the board. This mechanism was activated when the managers tried to obtain the Executive Board’s acknowledgment by presenting new ideas, expressing concerns or when they critically challenged the performance of the other managers and their management styles with the goal of gaining acknowledgment and respect from the Executive Board.

Thus, the context that triggered the mechanisms of staging was the presence of the Executive Board. The presence of the board at the oilcloth sessions constituted a social context that activated the managers to stage their perspectives and concerns regarding the implementation of the new ED. Having the Executive Board present at all oilcloth sessions motivated the participants to express themselves, resulting in the participants’ experience of being respected. Therefore, we find that when the oilcloth sessions work as a stage, the managers experience being involved and that their opinions are taken seriously, a ripple effect that may increase the acceptability of the implementation process.

### A battlefield

Counter the proposed program theory, participation and involvement did not necessarily lead to a positive view of the oil cloth sessions and this ripple effect thus represents a negative unplanned outcome. Most of the participants characterized the oilcloth sessions as a battlefield. This became evident through the facilitator’s use of militaristic metaphors: “You should see it as if you are standing at the front line ready to receive” [informant 51] or “Please stand by, just like [we are] playing battleships” [informant 51]. The use of war metaphors affected communication at the oilcloth sessions, which meant that several of the participants started referring to the Executive Board as “the Fuehrer Bunker” [fieldnotes no. 4]. For some of the participants, these war metaphors were perceived as uncomfortable and frustrating:

It was uncomfortable and inappropriate to use words from war, what was it for? It created a negative climate. [informant 045]

One area that appeared as a battle was whether the Executive Board sincerely wanted the participants’ involvement or whether it was just a tokenistic involvement understood as based on a false appearance of engagement. In relation to a discussion on the number of patient beds in the new ED, a manager expressed:

Then suddenly, we are not involved, just like that. Will there be room for us at all? Then it ends up that we suddenly stood and fought, right? [informant 007]

Participants experienced “professional battles” between the managers from the specialist departments and the managers from the existing and the new ED. These battles were marked by a lack of confidence in the other specialty’s handling of the patient pathways:

Then this professional war appeared, yes specialist war. In any case, I think it suggested […] a lack of confidence in the different [specialties] being able to make the right decisions [informant 011].

Finally, for participants from one of the surgical departments, the oilcloth sessions disclosed that the implementation of a new ED would not be a merger between departments but a subdivision with winners and losers. One manager expressed:

Then we come back to it again, merger versus division of departments. Especially when it comes to how we as physicians should work. There you get a lot of that underlying perception. It gives the feeling that it is us who lose, and they win. [the managers from the ED] [informant 021]

Oilcloth sessions provided a space where battles of involvement, influence, and specialty were fought, leading to mistrust of each other and each other’s intentions and professionalism, which again seemed to decrease participants’ acceptance of the upcoming implementation process.

The mechanism of the battlefield in the oilcloth sessions was also triggered by organizational uncertainties about the physical environment of the new ED and how emergency care tasks would be allocated. Among other things, the building had yet to be finished. Furthermore, the new specialty of emergency medicine was to be merged into the new ED together with the other specialties, resulting in new allocations of the tasks in emergency care. These factors posed a possible threat to the different specialties involved in emergency care. Bringing the specialties and the Executive Board together at the oilcloth sessions triggered the use of war metaphors among the participants to describe how they fought and protected their area of specialty during the sessions. Thus, we argue that these organizational uncertainties provided a battle about influence and involvement, which triggered the managers to fight for and defend their medical specialty during the oilcloth sessions, increasing mistrust of the implementation process among the managers. Ultimately, this ripple effect may diminish the planned implementation process. One manager expressed:

I have become completely unsure whether the implementation process is under control at all, after having participated in the oilcloth sessions. I do not think it will be possible to implement the ED as described. [informant 032]

### A space for creativity

As an important addition to the proposed program theory, the analysis showed that oilcloth sessions created time for reflexive and creative thinking, which increased managers and key employees’ motivation to engage in the implementation of the new ED. Regardless of whether the participants worked with existing or new patient pathways in the oilcloth sessions, the question of “what if?” became central for the development and collaboration among the participants throughout the sessions. The question of “what if” opened a space for creativity defined as the use of original ideas and imagination, understood as the capacity to create, evolve, and exploit mental models of things or situations that do not yet exist (Stevenson, 2003). Unlike the managers’ daily busy practices, with little time for reflection, oilcloth sessions became a space that allowed reflexive thinking free from disturbances. A manager from the other departments exclaimed: “Great to have time to just be in one place” [informant 11]. Reflexive thinking became a mechanism for imagining new opportunities, creating better ideas such as new patient pathways:

Imagine that upon the patient's arrival at the ED, we can provide a unified, interprofessional team that can clarify the patient's further course within a short time. [informant 035]

Other ideas were linked to collaborations regarding new ways of working together and organizing work in the new ED. For some participants, oilcloth sessions became an opportunity to perform possible future work procedures:

It became a bit of a theatre for how we can imagine it might become in the future. I do not know if our ideas are going to be realized. But participating provides a lot of inspiration. [informant 009]

Although some of the staff and key employees knew about the physical layout before participating in the oilcloth sessions, they were surprised about how the layout created uncertainties and worries about the future. A participant expressed:

Even though we have received drawings and seen how the room distribution is, we were still surprised to see how far you have to walk and where to sit. Then you create negative ideas … how will it work? We have tried to change practice so many times, why should we succeed this time. [informant 005]

Thus, oilcloth sessions also became a space for negative thoughts about the future.

Even though some participants were skeptical about the advantages of playing with Lego figures on an oilcloth, we found that oilcloth sessions were generally perceived as a space that allowed imagination, improvisation, and inspiration.

Time is scarce in emergency care. Time away from their busy routine created an opportunity for the participants to engage in reflexive thinking without being disturbed. We argue that time free from daily disturbances allows managers and key employees to create better ideas and become motivated to engage in the implementation of the new ED. This ripple effect thus adds that time free from disturbances may increase the acceptability and quality of the implementation process.

### A strategic management tool

The analysis shows that oilcloth sessions emerged as a strategic management tool before the sessions even took place. Thus, the oilcloth sessions set in motion reactions even before the implementation strategy took place because the managers saw a possibility to position themselves as managers towards the employees in their department. This chain of reaction of events bring important knowledge to the proposed program theory about how the strategy affects reactions and reasoning beyond the strategy itself, and how these may be conducive to the successful operation of the implementation strategy.

Some managers gave much consideration about which staff members to invite to the oilcloth session and why. A manager explained:

There has been opposition to the new ED and that's why I brought a physician who had negative thoughts against the new ED with me to the session to make him see the possibilities, right? I used it as a managerial tool. [informant 46]

By inviting physicians who had a negative attitude towards the new ED, oilcloth sessions emerged as a strategic management tool for managers to break down resistance in relation to the implementation of a new ED. One manager expressed:

Before our participation, I tried to think and plan strategically who I would invite from my department. [informant 18]

Other managers anticipated disagreements between the participants from the specialist departments and the ED about, for example, the organizational plan. The managers tried to overcome this resistance by bringing formal policy documents to the oilcloth sessions, which they could refer to in the discussions. They strategically prepared counter arguments before participating in the sessions.

Some managers invited key employees to participate because they believed it important that were seen to “fight” for their specialty vis-à-vis the Executive Board. They positioned themselves as a strong manager who stepped into character at oilcloth sessions and visualized their management mandate to the employees. A manager expressed:

I want to show my staff that I am a fighter. I will do anything to secure our patients. That is what a strong manager does. [informant 15]

Besides engaging in the planning of the implementation of the new ED, the oilcloth sessions provided an opportunity for the department managers to position themselves as “strong management and managers”, primarily towards the employees in their own department as well as the other department managers and the Executive Board. This response to the oilcloth sessions was activated not only at the sessions but also as part of the preparatory work before the sessions. The employees in the individual departments constituted the social context that triggered department managers to use the oilcloth sessions to display their management skills. Oilcloth sessions, as a strategic tool to manage employee’s perceptions of the new ED, activated managers to position themselves as strong managers, engaging strategically in the implementation of the new ED. The overall outcome of this ripple effect may positively influence the acceptability, effectiveness, and sustainability of implementation, although the reasoning alluded to a different aim: the individual managers’ position as leader.

## Discussion

The results show that the oilcloth implementation strategy activated mechanisms that led to four ripple effects: a stage, a battlefield, a space for creativity, and a strategic management tool. Overall, we found that these ripple effects affected the implementation outcomes both negatively and positively. One positive effect was that, when the oilcloths “work” as a stage, the managers experience being heard, listened to, and involved by the Excusive Board, which may have increased acceptance of the implementation process as intended. However, a scene did not only have a positive effect. When the Excusive Board acknowledged some managers, other managers in the room might have felt overlooked or a need to assert themselves towards the Excusive Board. Thus, the stage became entwined with a battlefield, which had a negative impact on the implementation process. Thus, the results indicate activation of mechanisms that led to ambiguous or negative ripple effects, depending on the participants’ reasoning and social dynamics (e.g. power). One example is how the presence of the Executive Board could obstruct the progress of the educational strategy, because oilcloth sessions became a battlefield of interests instead of a “safe” place with room for education, innovation, and thoughtfulness. Because we find that participation in oilcloths sessions does not unambiguously lead to a positive attitude towards the implementation object a refinement and improvement of the initial program theory is needed. Thus, for future studies of oilcloth sessions, we recommend paying attention to potential negative attitudes generated by participation in oilcloth sessions. Whether the ripple effects we have identified in this study may be generic to other collaborative implementation strategies or decision models requires further research. Contrary to our findings, a recent study exploring how an action learning program could improve the mental health promotion capacity on organizational level, showed that the implementation strategy strengthened the inter- and intra-organizational collaboration and thus did not find negative attitudes generated by participation (Hinrichsen *et al*., 2022). However, the participants in the implementation strategy in the Hinrichsen study differed from our study, because they came from three different local organization and were either employees, students or volunteers. A reason for the ripple effects found in our study may thus be a result of the participants in the oilcloth session had different roles and organizational power and worked in the same organization.

The use of war metaphors activated a sense of urgency and even anxiety, which may motivate further action and become an effective way of grabbing the participants' attention (Flusberg *et al*., 2018). Although war metaphors are often used in public discourse, because they tap into basic and widely shared schematic knowledge that efficiently structures our ability to reason about any situation involving opposing sides (Flusberg *et al.*, 2018), their use in the oilcloth sessions became counterproductive for many of the participants. According to Le Guin (2012), the consequence of a facilitator using war metaphors is a division between the participants into Them (bad) and Us (good) which creates mistrust. Thus, instead of creating a sense of community and the experience of being on what Robins and Mayer (2000) called a two-way street, the use of war metaphors led to a combative way of viewing the future, which for some participants reduced the oilcloth sessions to a battlefield and future cooperation and the implementation of a new ED became a war. Facilitators of future oilcloth sessions should be attentive to articulation of an implementation as a war, and the ways in which it is not, if the ripple effect of “a battlefield” is to allow the participants to experience that they were on a joint journey of implementing a new ED rather than a negative outcome.

The study also shows that the use of oilcloth sessions as an implementation strategy became a space imbued with interpretations of current practice and visions about the future, expressed as imaginative ideas. For some participants, these visions of the future were viewed positively, meaning that the participants experienced that collaboration in the oilcloth session created the opportunity to come close to the goal of implementing the new ED. For others, these ideas became a barrier to reach the goal. Ideas help create a shared imaginative horizon, understood as an available pool of resources (Hasse and Søndergaard, 2020) that form what Lave and Wenger (1991) called “a community of practice”. This community of practice provided certain understanding about the future of the new ED through shared activities in the oilcloth session. For the participants leaving the oilcloth sessions with a positive view of the future implementation, the goal from the Executive Board was fulfilled (see the program theory); however, for participants leaving the oilcloth session with a negative view of the future implementation of the new ED, the strategy failed.

In line with Poland *et al.* (2008), we found that the context of the implementation process, the new ED, influenced how the implementation strategy emerged. Organizational changes are ongoing in the healthcare sector to achieve sustainable growth and survival, thus managers and implementation champions are expected to implement change all the time (Chung *et al*., 2017). However, the organizational uncertainties about the implementation of the new ED created mistrust among the participants at the oilcloth session, which in turn could decrease the acceptance of the new ED. Although it is recognized that unintended outcomes of social programs might affect the development, implementation, and evaluation of public health interventions (Bonell *et al*., 2015), these are rarely addressed in the literature (Lorenc and Oliver, 2014) and implementation research. Lorenc and Oliver (2014) identify five potential harms of public health interventions and argue that in interventions involving behavior change, unintended outcomes are difficult to identify and illustrate. Bonell *et al.* (2015) suggested using Dark Logic Models to identify potential unintended outcomes of interventions. The increasing attention to unintended outcomes originates from a critical view of a too narrow focus on the positive gains of the intervention change (Broholm-Jørgensen, 2022) and researchers’ hope that the interventions will work. Implementation strategies resemble public health interventions in the sense that both concern activities of change in complex systems based on underlying assumptions of feasibility and effectiveness. Focusing on unintended outcomes in implementation research could help us anticipate negative unintended outcomes or identify unintended consequences that could be leveraged or addressed in future implementation efforts to enhance outcomes, effectiveness and sustainability (Bonell *et al*., 2015; Pullmann *et al*., 2022). There is a need within implementation research to incorporate attention to unintended outcomes to fully understanding the impact of implementation strategies. In our view, this attention is best qualified by the use of ethnographic methods combined with interviews, contributing knowledge about how, for whom and under what circumstances an implementation strategy works (Gertner *et al*., 2021; Kirk and Haines, 2020).

Although it may be important to select strategies that can overcome defined determinants and make the expected mechanisms behind them explicit to ensure and support a successful implementation (Powell *et al*., 2019), the question remains, to what extent the application of implementation strategies in clinical practice can be determined before and outside the situational context in which they operate? Causal mechanisms of implementation strategies can be perceived as linear, with context sliding into the background as a static factor or simply as a physical locality (Nilsen and Bernhardsson, 2019); however, we know that reality is much more complex and that we should strive to model that complexity to the extent possible.

In this study, we define ripple effects as unintentional, unplanned, unanticipated outcomes that are caused by an implementation strategy (Pullmann *et al*., 2022). However, in some realist evaluation studies, the concept of ripple effects is applied to illustrate how intervention activities generate series of (sequential) CMO configurations (Jagosh *et al*., 2015). In implementation research, the concept of a series of events in a system could help understand how implementation strategies accumulate in sequences, with mechanisms or outcomes of one sequence informing or transforming the context of the subsequent sequences. For example, the managers' mistrust in the implementation strategy, which was identified as leading to a decrease in fidelity of the implementation process, may be an important contextual factor for how the employees’ reason in the future implementation process. The mechanism identified in one stage of the implementation process may inform or transform the context of subsequent stages. This also includes CMO configurations as series of events that provide the possibility to explore how an implementation strategy influences implementation as it progresses. We suggest future studies of implementation strategies include analytical attention to series of CMO configurations of unintended and intended outcomes to explore longitudinal outcomes of implementation strategies.

### Strengths and limitations

By identifying the underlying mechanisms of how oilcloth sessions “work” in the context of implementation of a new ED, we provided a compilation of possible ripple effects linked to the oilcloth implementation strategy, which in turn can improve the quality and impact of the implementation (Lipsitz, 2012). We are aware that other ripple effects were at play, such as tokenism of participants, but as part of our analytical process, we selected the ripple effects that most predominant in the data material. The use of a realist evaluation approach is particularly applicable when exploring how complex systems, such as implementation strategies, work, and to identify unintended outcomes that may interfere with achieving the intended outcomes. Although identifying context is a key element of the realist evaluation approach, defining the context and separating mechanisms from the context is difficult because any changes among the participants, the organizational context or the overall implementation can affect the implementation outcomes (Dossou *et al*., 2021). Based on this perspective, an implementation strategy will not have the same outcome in every target group or in any institutional context or infrastructure, and changes in a particular context may trigger other mechanisms than identified in a study. We argue that the findings in this study are still relevant in implementation research, because they illustrate how a well-defined implementation strategy also produced unexpected outcomes. Furthermore, the study highlights important contextual factors relevant for effective implementation of organizational changes, such as organizational uncertainty and time. The importance of context is widely recognized in implementation and evaluation research (Dossou *et al*., 2021; Poland *et al*., 2008), however, the realist evaluation approach in this study contributes with a focus on context that transcends the implementation strategy by revealing unintended ripple effects caused by mechanisms triggered by the existing contexts.

There is consensus in the field of implementation research that implementation is likely a recursive process with well-defined stages that are not necessarily linear and that have an impact on each other in complex ways (Fixen and Blase, 2009), therefore one potential limitation is that we examined the oilcloth strategy within a limited timeframe/phase of implementation according to QIF phase 1 (Meyers *et al*., 2012); examining the strategy over several implementation phases could provide the opportunity to see if this strategy has an interesting influence as implementation of the ED moves ahead.

The implementation of a new organizational model requires the cooperation and permission of gatekeepers who control access to certain settings and potential participants (Poland *et al*., 2008). In this study, we found that the managers use of the oilcloth sessions as a strategic tool had implications on which participants participated in the oilcloth sessions. This dual role as gatekeeper and manager may have intentionally or unintentionally influenced the discussion in the oilcloth sessions and how the participants voiced their satisfaction with the new ED at the sessions and thereby the data available in this study.

## Conclusion

We have identified four ripple effects of oilcloth sessions that seemed to influence targeted implementation outcomes and further the implementation process. The results provide important knowledge that may help explain how and why a well-defined implementation strategy has unplanned outcomes. Attention to unintended outcomes of implementation strategies may help implementation researchers rethink the activities involved to reduce negative unintended outcomes or leverage positive unintended consequences to enhance implementation and health outcomes. We suggest future studies within implementation research incorporate attention to unintended ripple effects and outcomes to fully understand the impact of implementation strategies (Pullmann *et al*., 2022)

## Figures and Tables

**Table 1:** Programme theory for the oilcloth sessions

	If	Then	Intended outputs	Intended outcomes
Oilcloth sessions	If the managers and key employees participate in the oilcloth session	Then they will develop their knowledge and learn about the new ED and change their attitude in relation to the new ED	If the managers and key employees change their attitude in relation to the new ED	Then the managers and key employees will respond more positively to the impending implementation of the new ED = increase in acceptability
	If the managers and key employees participate in the oilcloth session	Then they will have the experience of getting involved and get a positive view of the sessions as a useful strategy when implementing a new ED	If the managers have the experience of getting involved and become positive towards oilcloth sessions as a strategy	Then their ownership for participating in oilcloth sessions will increase and they will take responsibility for implementing the new ED = increase in acceptability
	If the managers and key employees participate in the oilcloth session	Then they will gain knowledge and insight into the new physical and material context	If the managers and key employees gain knowledge and insight into the new physical framework, then their experience of sense of place will increase, contributing to a positive experience towards the new ED	Then their motivation for supporting the implementation of a new ED increases = increase in acceptability

**Source(s):** Authors’ work

**Table 2:** Participating departments

Specialty	Department
Medical specialty	Cardiology Gastroenterology (Medical) Infectious Diseases Internal Medicine (including Respiratory Medicine and Endocrinology)
Surgical specialty	Orthopedic Surgery Gastroenterology (Surgical)
Emergency specialty	Emergency Department
Other	Clinical Biochemistry Obstetrics and Gynecology Paediatrics and Adolescence Medicine Radiology

**Source(s):** Authors’ work

**Table 3:** participants in the interviews

Number (*N* = 53)	Profession and positions
26	Physicians (10 chief physicians, 13 senior physicians and three trainee physicians)
19	Registered nurses (8 head nurses, 8 charge nurses, 1 assistant charge nurse, 1 clinical nurse specialist and 1 registered nurse)
1	Head midwife
2	Managing medical secretaries
2	Bioanalyst (1 bioanalyst and 1 chief bioanalyst)
1	Charge radiographer
2	Members of the Board of Directors

**Source(s):** Authors’ work

**Table 4:** Identified CMO configurations

Ripple effect	Context	Mechanism (resource and reasoning)	Outcome
A stage	The presence of the Executive Board at the oilcloth sessions	A possibility to present new ideas and concerns are heard and acknowledged (resource) → The managers experienced being involved, and their opinions were taken seriously (reasoning)	Increased commitment and acceptability of the implementation process
A battlefield	Uncertainty in the implementation of the new ED	A battle for influence and involvement (resource) → Mistrust among managers in the implementation process (reasoning)	Decreased fidelity of the planned implementation process
A space for creativity	Time free from disturbances	Reflexive and creative thinking (resource) → Managers and key employees motivated to engage in the implementation of the new ED (reasoning)	Increase in new and better ideas and increased acceptability and quality of the implementation process
A management tool	Oil cloths sessions	A possibility for management positioning (resource) → Managers engage strategically in the implementation of the new ED (reasoning)	Positive influence on implementation acceptability, effectiveness, and sustainability of the implementation process

**Source(s):** Authors’ work

**Table A1:** Sub-themes and themes

Sub-themes	Themes
A mouthpiece	A scene
A showroom	
An arena with different agendas	A battle arena
An arena of tokenistic involvement	
A battle and position war	
Winners and losers	
Expansion of imagination	Imaginations
Creating an imagination of instability	
Imaginations about physical spaces and settings	
imaginations about the future collaboration and organization	
Imaginations about the specialties fate in a future new ED	
Fictional world	
Documents are used for verification for disagreement	Management tool
disciplining space (disciplinerende rum)	
Management strategic tool	

**Source(s):** Authors’ work

## Appendix 1 A picture of the oilcloth session

Table A1
